# A young girl with severe polyarteritis nodosa successfully treated with tocilizumab: a case report

**DOI:** 10.1186/s12969-021-00654-7

**Published:** 2021-12-03

**Authors:** Margaux Boistault, Mireia Lopez Corbeto, Pierre Quartier, Laura Berbel Arcobé, Ariadna Carsi Durall, Florence A. Aeschlimann

**Affiliations:** 1 Department of Pediatric Immunology-Hematology and Rheumatology, Necker University Hospital – Assitance Publique-Hopitaux de Paris, Enfants Malades,149, rue de Sèvres, 75015 Paris, France; 2 Pediatric Rheumatology Unit, Vall d’Hebron Hospital Campus, Barcelona, Spain; 3 Université de Paris, Imagine Institute, Paris, France; 4 Rheumatology Department, Mútua de Terrassa University Hospital, Terrassa, Barcelona Spain; 5 Pediatric Hospitalisation Unit, Vall d’Hebron Hospital Campus, Barcelona, Spain

**Keywords:** Polyarteritis nodosa, Child, Tocilizumab

## Abstract

**Background:**

Childhood Polyarteritis nodosa (PAN) is a systemic vasculitis with necrotizing inflammation of medium- and small-sized arteries. Disease evolution may be severe and refractory to standard treatment including prednisone, azathioprine and cyclophosphamide.

**Case presentation:**

We present the case of a young girl with severe PAN resulting in progressive ischemia and necrosis of fingers and toes. Biological work-up revealed increased acute phase reactants and interleukin-6 levels. She was only partially controlled despite high-dose corticosteroids and cyclophosphamide infusions, and eventually achieved rapid improvement and sustained remission on tocilizumab.

Further, we review the current evidence of the interleukin-6-inhibitor tocilizumab for the treatment of PAN.

**Conclusion:**

Tocilizumab may be an efficient therapeutic option in a subset of treatment-refractory children with PAN.

## Background

Polyarteritis nodosa (PAN) is a rare systemic vasculitis with necrotizing inflammation of medium or small sized arteries that may start in childhood [1]. The presentation and clinical course can be variable and range from relatively benign to severe systemic forms affecting multiple organs. In childhood, PAN relapse rates have been described up to 50%, therefore morbidity and mortality are non-negligible [2, 3]. Treatment recommendations include high-dose corticosteroids and cyclophosphamide for severe disease; however, their long-term use may be associated with unfavourable adverse events, especially in children. In recent years, biological agents including TNF-inhibitors (infliximab, adalimumab) and rituximab have been used for refractory cases [4, 5]. Here, we describe the use of tocilizumab (TCZ), an interleukin-6 (IL-6) receptor inhibitor, in a little girl with severe, refractory, necrotizing PAN. Informed parental consent was obtained for the publication of the case and the images.

## Case presentation

**Fig. 1:**
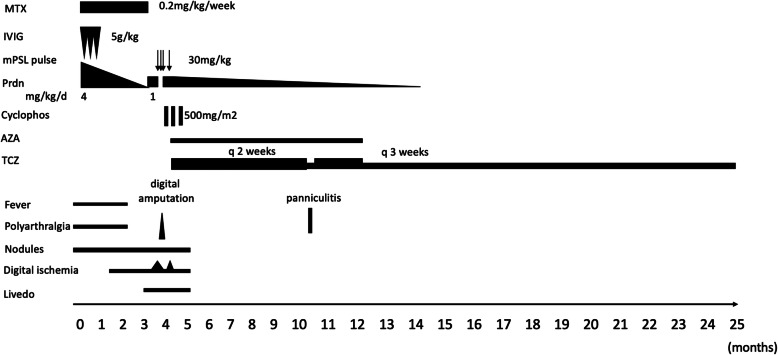
Clinical course and treatment regimens. AZA = Azathioprine. Cyclophos = Cyclophosphamide. IVIG = intravenous immunoglobuline. mPSL pulse = Methylprednisolone pulse. MTX = Methotrexate. Prdn = Prednisone. TCZ = Tocilizumab

A previously healthy 4-year-old girl of North African origin developed fever, polyarthralgia, swelling of her hands and feet, palpable nodules in legs, cyanosis and progressive ischemia of the second to fifth right fingers and the second right toe. There was no family history of immunodeficiency, autoimmune or vascular disease. She initially presented at a local hospital in North Algeria where a skin biopsy from a leg nodule was performed showing a vasculitis of the medium-sized vessels suggestive of polyarteritis nodosa. Therapy with high dose prednisone at 4 mg/kg/day, three immunoglobulin infusions at 5 g/kg and methotrexate at 0.2 mg/kg/week was initiated without improvement. The authors do unfortunately not have more information about the rationale for choosing these high doses of prednisone and immunoglobulin infusions. Disease course and treatment regimens are shown in Fig. 1.

Three months later parents sought a second opinion in Spain due to worsening disease. At initial presentation the girl was well and afebrile, blood pressure was 98/72 mmHg. She had cutaneous necrosis of the distal phalanx of the second to fifth fingers of the right hand and the second toe of the right foot, palpable nodules in lower limbs without livedo reticularis and myalgia. Pulmonary, abdominal, ear-nose-throat and neurologic exams were normal, there was no evidence of neuropathy.

Laboratory investigations showed elevated acute phase reactants (C-reactive protein 100 mg/l (normal < 5 mg/l), erythrocyte sedimentation rate 120 mm/h, and increased pro-inflammatory cytokines (IL-6 106.43 pg/ml (normal 0–4.3 pg/ml), IL-10 9.62 pg/ml (normal 0–7.8 pg/ml), TNF-alpha 10.7 pg/ml (normal 4–8 pg/ml), measured by ELISA). Creatine kinase was within normal limits, as were liver transaminases. Hepatitis B serology showed immunity through vaccination. The autoimmune workup was negative including anti-phospholipid antibodies.

**Fig. 2:**
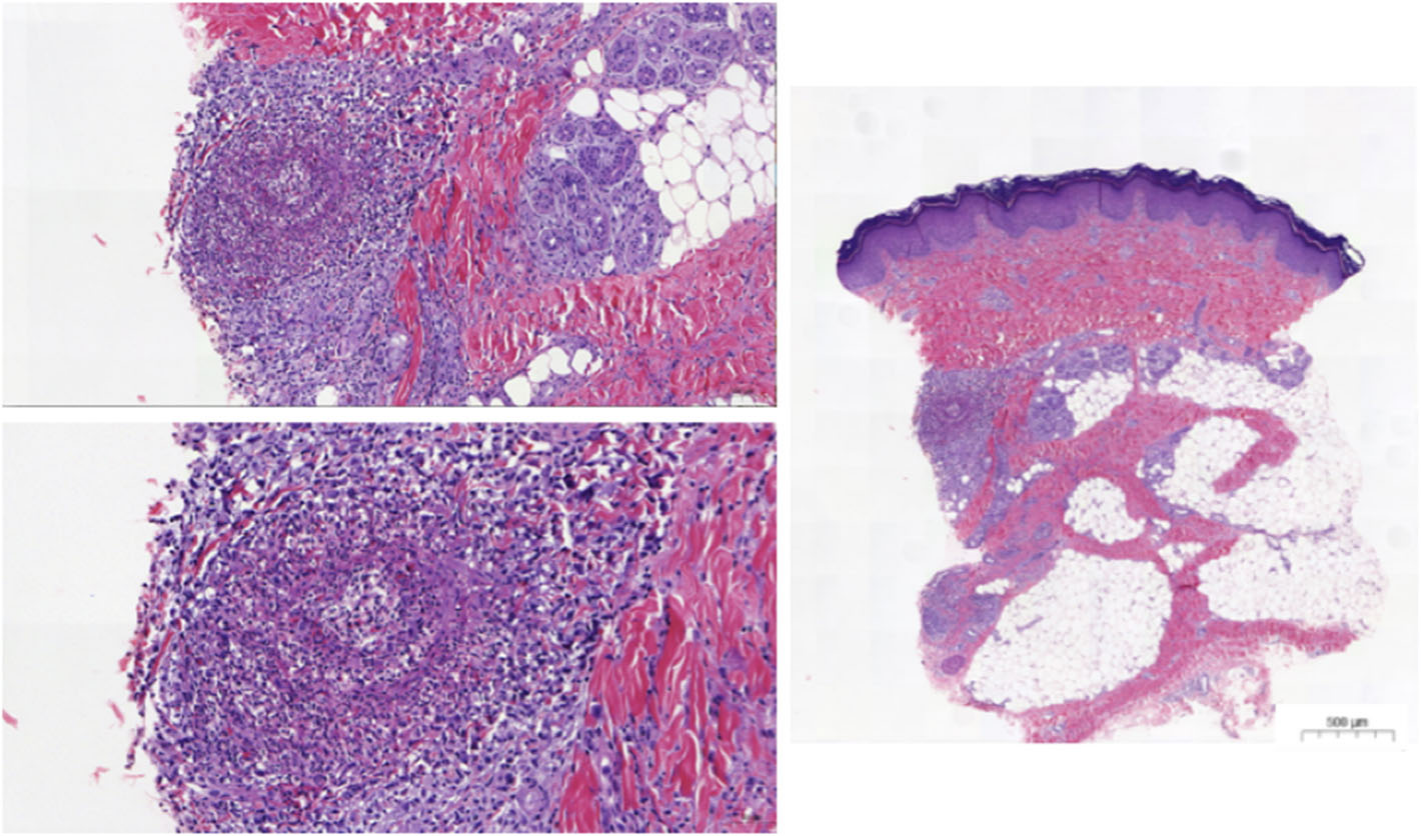
Histology from skin biopsy. Skin biopsy from a nodule on the foot sole. Histology (hematoxylin eosin staining, × 10 magnification) demonstrated inflammation of a small artery with an inflammatory infiltrate of predominantly lymphocytes with neutrophils. No granulomas were seen

Broad microbiologic cultures were negative and no anti-tissue specific antibodies were found. A primary immunodeficiency panel of 323 genes (including *CECR1* for ADA2 deficiency) did not find any pathogenic mutation. Positron emission tomography scan revealed muscular, synovial and diffuse bone involvement, with hyperfixation also of lymph nodes and subcutaneous nodules. A second skin biopsy was performed from the sole of foot and compatible with PAN (Fig. 2). Muscle biopsy was normal.

**Fig. 3:**
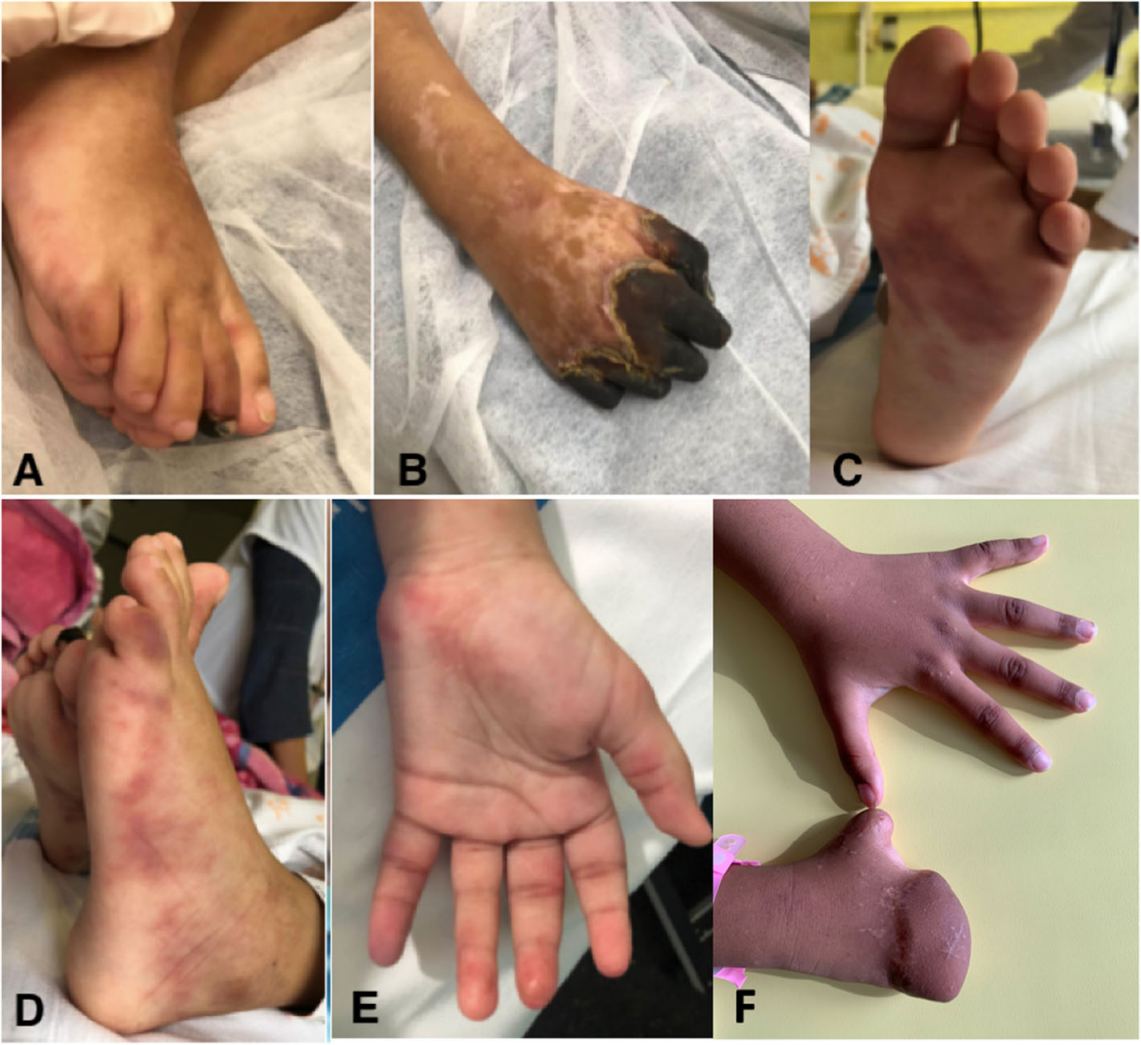
Ischemia of the extremities. **A**, **B** Cutaneous necrosis of the distal phalanx of the right hand and the second right toe prior to surgery. **C**-**E** Vasculitic lesions of the feet and left hand. **F** Right hand, 21 months after treatment initiation

Intravenous corticosteroids were initiated at 1 mg/kg/day for 8 days, but disease activity persisted and the patient was given methylprednisolone 30 mg/kg/day on three consecutive days. However, disease was still not controlled and cutaneous necrosis required amputation of the 2nd to 5th finger of the right hand (Fig. 3). Cyclophosphamide at 500 mg/m^2^ dose and subsequently azathioprine at 1 mg/kg/day were started due to worsening peripheral oedema, subcutaneous nodules and painful livedo. Despite of treatment escalation and low-dose aspirin, new ischemic signs appeared on the 5th finger of the left hand for which intravenous infusion of alprostadil at 9.6 ng/kg/minute that was maintained for 5 days, a second dose of cyclophosphamide and another bolus of methylprednisolone 30 mg/kg were given. Given the partial response, persistent biologic inflammation and high serum of IL-6 levels, TCZ (8 mg/kg every 2 weeks) was added with clinical and biological improvement within few days. Azathioprine was continued and Prednisone slowly tapered. Six months after TCZ initiation, after increasing the TCZ infusion interval to 3-weekly, she developed panniculitis; TCZ infusion was again administered in 2-weekly intervals while Azathioprine was continued, and panniculitis improved. After the child had moved to France in complete remission and 12 months after treatment initiation, TCZ infusions interval was increased to every 3 weeks and azathioprine discontinued. Prednisone was discontinued after 14 months. At last follow-up, 21 months after treatment initiation, complete clinical and biologic remission persists on 3-weekly TCZ infusions and low-dose aspirin. Serum IL-6 level remained elevated (43.3 pg/ml, normal 0.0–8.5). Serum ADA2 activity was normal. Thoracic and abdominal CT angiography showed no abnormalities.

## Discussion and conclusions

We hereby report rapid improvement and sustained remission following TCZ therapy in a little girl with severe, treatment-refractory PAN. TCZ was favoured over a TNF-blocker due to high acute phase reactants (indicating increased inflammation) and markedly elevated IL-6 levels (relatively higher compared to TNF-alpha level elevation). Indeed, TNF-inhibitors may be the primary choice in patients in which ADA2 deficiency cannot rapidly be excluded as was the case in our patient by negative genetic testing and normal serum ADA2 activity; TNF-inhibitors have been successfully used in children with PAN and ADA2 deficiency [6, 7].

TCZ was well tolerated without serious adverse events during the 18 months follow-up period. Prior to TCZ, the patient had received high-dose corticosteroids, cyclophosphamide and azathioprine; therefore, a cumulative beneficial effect of these medications cannot be excluded. However, we believe that TCZ was responsible for the clinical and biological improvement observed in the patient because disease activity worsened despite treatment escalation, there was a marked and fast improvement after TCZ initiation and the patient has maintained a sustained clinical remission with exception of a mild disease flare when TCZ dose interval was temporarily increased.

To date, eleven patients (median age 35 years, IQR 23.5–57.5; 5 female) with PAN treated with TCZ mostly for refractory disease have been reported in the literature [8–13]; amongst these, two children (Table 1). Watanabe et al. published the case of a 3-year-old boy presenting with fever, periostitis, epididymo-orchitis, arthritis and myositis, who improved clinically, biologically and radiologically on TCZ and cyclophosphamide after having failed IVIG, corticosteroids and infliximab [9]. Inoue et al. reported an 8-year-old girl with cutaneous PAN resistant to multiple treatment lines including corticosteroids, azathioprine and TNF-inhibitors, who eventually achieved remission on TCZ [8]. In adults, TCZ has been successfully used in eight treatment-refractory cases [10–14] and in one patient as first-line therapy in combination with corticosteroids [10]. Our patient received intravenous tocilizumab at a dose of 8 mg/kg every 2 weeks in analogy with the dose used by Inoue an colleagues [8]. Moreover, this lower dose was chosen due to an increased risk for potentially severe infections associated with the therapy, as tocilizumab was initiated only a few days after digital amputation. Later the dose was maintained as clinical and biologic response to tocilizumab was rapid and excellent. Various dosing regimens and administration routes (subcutaneous and intravenous) have been used in the literature, and the optimal dosing regimen still needs to be determined.

**Table 1:** Overview of published PAN patients treated with tocilizumab

Reference	Age/Sex	Symptoms	CRP (mg/l)	Previous treatment TCZ treatment	Outcome
Inoue [8]	8y/F	cPAN, fever, nodules, arthritis	100	GC, AZA, cyclo, MMF, tacrolimus, IVIG, anti-TNF TCZ 8 mg/kg q2 wks IV	GC stopped Remission (FU 7 mo)
Watanabe [9]	3y/M	Fever, periostitis, arthritis, myositis, epididymo-orchitis	200	GC, IVIG, IFX TCZ 10 mg/kg q4 wks + CYC	GC tapering Improvement (FU 6 mo)
Krusche [10]	23y/M	Livedo racemosa, myalgias, fever, weight loss, sensorimotor polyneuropathy, sc nodules	291	GC, MTX, RTX, CYC, ANR, IVIG TCZ 8 mg/kg q4 wks IV + IVIG + GC, later TCZ 10 mg/kg q4 wks IV	Low dose GC Remission (FU 37mo)
Krusche [10]	24y/M	Myalgias, fever, weight loss, arthritis, sc nodules, drop hand, abdominal and flank pain, aHT, increased creatinine (1.3 mg/dl)	298	GC, IVIG, CYC TCZ 8 mg/kg q4 wks IV + urbasone IV, later TCZ 162 mg weekly SC	Low dose GC Remission (FU 11mo)
Krusche [10]	63y/F	Myalgias, fever, fatigue, weight loss	174	GC TCZ 162 mg weekly SC + GC, later TCZ 162 mg weekly SC	Low dose GC Remission (FU 6mo)
Krusche [10]	70y/F	Myalgias, arthritis, livedo racemosa, sensorimotor polyneuropathy	93	GC, MTX TCZ 8 mg/kg q4 wks IV + GC, later TCZ 8 mg/kg q4 wks IV	Low dose GC Mild livedo racemosa, otherwise asymptomatic (FU 13mo)
Saunier [11]	39y/F	Necrotic purpura, myalgia, weight loss	59–126	GC, MTX, MMF, CYC, IFX, AZA TCZ 8 mg/kg q4 wks IV	Low dose GC Remission (FU 12mo)
Saunier [11]	52y/F	Necrotic purpura, livedo, ulcerations, myalgia, arthritis, neuropathy, weight loss		GC, DAP, COL, MTX, CYC, AZA TCZ 8 mg/kg q4 wks IV	Low dose GC Remission (FU 12mo)
Saunier [11]	35y/M	Arthritis, myalgia, tenosynovitis, nodules, weight loss	393	GC, IVIG TCZ 8 mg/kg q4 wks IV	GC stopped Remission (FU 10mo)
Hocevar [12]	33y/M	Ulcers, aHT, myalgia, weight loss, nodules	169	GC, CYC TCZ 8 mg/kg q4 wks IV	GC tapering Improvement (FU 50mo)
Bodoki [13]	67y/M	Fatigue, weight loss, myalgia, arthralgia, polyneuropathy	20	GC, CYC TCZ 162 mg weekly IV	Low dose GC Remission (FU 12mo)

*aHT* Arterial hypertension. *ANR* Anakinra. *AZA* Azathioprine. *COL* Colchicine. *cPAN* Cutaneous polyarteritis nodosa. *CYC* Cyclophosphamide. *Cyclo* Cyclosporine. *DAP* Dapsone. *F* Female. *FU* Follow-up. *GC* Glucocorticoids. *IFX* Infliximab. *IV* Intravenous. *IVIG* Intravenous immunoglobulins. *M* Male. *MMF* Mycophenolate mofetil. *MTX* Methotrexate. *RTX* Rituximab. *SC* Subcutaneous. *TCZ* Tocilizumab. *Q4 weekly* every 4 weeks. *Y* Years

Whilst mutations in *CECR1* are associated with monogenic PAN in some patients, the etiopathology of classic PAN is not well known [15, 16]. However, both the innate and adaptive immune systems seem to be involved [17]. Our and others clinical observations suggest a role of the pro-inflammatory cytokine IL-6 in the pathophysiology but very few studies have investigated this hypothesis [18–21]. Increased IL-6 levels have been associated with disease activity in some case reports [18, 20, 21], where IL-6 levels normalized with response to treatment (prednisone, cyclophosphamide, tacrolimus). In addition, Kawakami and colleagues retrospectively evaluated 45 patients with cutaneous PAN [19]. Those with elevated IL-6 levels (*n* = 19) were more commonly males, had significantly more arthralgia and skin ulcerations, higher CRP serum levels and higher prevalence of antiphospholipid antibodies compared to those with normal IL-6 levels (*n* = 26). However, none of these patients was treated with an IL-6 inhibitor and repeated IL-6 serum levels were not available [19].

In conclusion, our observation and the published literature suggests that TCZ may represent an effective therapeutic option in a subset of patients with recalcitrant PAN. However, many questions remain to be answered, in particular with the exact pathophysiological role of IL-6 in PAN which has yet to be elucidated, also how to determine which patients will respond to IL-6 blockade, and whether it should be considered as second-line treatment for refractory patients or as first-line therapy.

## Data Availability

Not applicable.

## Abbreviations

ADA2: Adenosine Deaminase 2
CECR1: Cat Eye Chromosome Region 1
IL: Interleukin
PAN: Polyarteritis nodosa
TCZ: Tocilizumab
TNF: Tumor necrosis factor
